# Exploring Community Succession, Assembly Patterns, and Metabolic Functions of Ester-Producing-Related Microbiota during the Production of *Nongxiangxing baijiu*

**DOI:** 10.3390/foods13193169

**Published:** 2024-10-05

**Authors:** Xiawei Yi, Huan Xia, Ping Huang, Shiyuan Ma, Chongde Wu

**Affiliations:** College of Biomass Science and Engineering, Sichuan University, Chengdu 610065, China; 2022223080027@stu.scu.edu.cn (X.Y.); 2021223085076@stu.scu.edu.cn (H.X.); 2021223080042@stu.scu.edu.cn (P.H.); 2023323085049@stu.scu.edu.cn (S.M.)

**Keywords:** fermentation grains, ester formation, microbial community, microbiota assembly

## Abstract

Esters are vital flavor compounds in Chinese *Nongxiangxing baijiu* and greatly affect the quality of *baijiu*. Microbial communities inhabiting fermented grains (FGs) have a marked impact on esters. However, the specific microorganisms and their assembly patterns remain unclear. This study utilized high-throughput sequencing and a culture-based method to reveal ester-producing microorganisms. A total of 33 esters were detected, including 19 ethyl esters, 9 linear chain esters, and 2 branched chain esters. A correlation analysis indicated that the bacterial genus *Lactobacillus* (relative abundance in average: 69.05%) and fungal genera *Pichia* (2.40%), *Aspergillus* (11.84%), *Wickerhamomyces* (0.60%), *Thermomyces* (3.57%), *Saccharomycopsis* (7.87%), *Issatchenkia* (0.96%), and *Thermoascus* (10.83%) were dominant and associated with esters production and their precursors. The numbers of esters positively correlated with them were 1, 17, 3, 2, 1, 1, 1, and 1, respectively. The modified stochasticity ratio (MST) index and Sloan neutral model revealed that bacteria were predominantly governed by deterministic processes while fungal assemblies were more stochastic. *Saturnispora silvae* and *Zygosaccharomyces bailii* were isolated and identified with ester synthesis potential. PICRUSt2 analysis showed that fungi in FG had a high potential for synthesizing ethanol, while 14 enzymes related to esters synthesis were all produced by bacteria, especially enzymes catalyzing the synthesis of acyl-CoA. In addition, ester synthesis was mainly catalyzed by carboxylesterase, acylglycerol lipase and triacylglycerol lipase. These findings may provide insights into ester production mechanism and potential strategies to improve the quality of *Nongxiangxing baijiu*.

## 1. Introduction

Chinese *baijiu*, a traditional alcoholic beverage made through spontaneous fermentation, ranks among the oldest distilled spirits in the world [1]. Due to its unique flavor, taste, and cultural deposit, *baijiu* has become the most well-known alcoholic beverage in China, boasting a history that spans more than two millennia [2]. *Nongxiangxing baijiu*, one of the four primary aroma *baijiu* types, is deeply loved by consumers for its strong cellar aroma, well-balanced and pure aroma, and lingering aftertaste. It represents approximately 70% of the market share [3]. *Nongxiangxing baijiu* (also called strong-flavor *baijiu*, SFB), mainly contributed by the regions of Sichuan, Heilongjiang, and Jiangsu, is brewed with grains as raw materials [4]. The grains, sorghum with or without glutinous rice, rice, corn, and wheat, are steamed and cooled before being mixed with *daqu*, which is then saccharified and fermented in the cellar called *jiaochi* [1,2]. Following that, raw liquor was produced by distilling the fermented grains (FG). During the fermentation process, carbohydrates, proteins, lipids, and other substances are degraded by enzymes produced by microorganisms, producing diversified flavor compounds [5]. The microorganisms in the fermentation system determine the profile of flavor compounds, and ultimately affect the overall quality of *baijiu*. Due to the application of varied and intricate materials, distinct production techniques, and the mixed-culture fermentation, the constituents of *baijiu* is extremely complex [6].

The composition, concentration, and varying ratios of different aromatic substances contribute to its special flavor, which is also the key factor influencing consumers to choose *baijiu*. Flavor analysis indicated that nearly 98% of the entire mass of *baijiu* was ethanol and water, and the remaining 2% was various flavor components [7]. Despite their low concentrations, these flavor components are pivotal in defining the aromatic profile of *baijiu*. Among them, ester is an extremely significant component of *baijiu* flavor, contributing to over 60% of the overall quality. Moreover, many esters make a great contribution to the smell, due to their low odor threshold, and affect the taste. Thus, the concentration and ratio of esters are one of the important criteria for the determination of *baijiu* quality [8].

Currently, the research on esters of *baijiu* mainly focuses on their variety, concentration, and variation during fermentation and aging [9,10,11,12]; their aroma contribution [13,14]; exploration and exploitation of ester-producing microorganisms [15,16]; and the mechanism of ester production [17,18]. For example, Fan et al. [15] isolated *Clavispora lusitaniae* from daqu, a yeast that demonstrated the ability to synthesize ethyl caproate. This showed its potential applications to generate flavor compounds to improve *baijiu* quality. A strain of *Aspergillus niger* was used by Xu et al. [19] for effective synthesis of fatty acid ethyl esters, and a possible mechanism for the catalytic process of esterification was proposed.

Lately, with advancements in high-throughput sequencing technology, numerous studies have emerged revealing the microbial composition and function through high-throughput technology combined with bioinformatics methods. The investigation of microorganisms, major flavor components, and their correlations in daqu, pit mud, and fermented grains has become a hotspot [20,21,22,23]. A total of 284 different esters have been detected in *Nongxiangxing baijiu,* and they are mainly ethyl esters. Microbial metabolism, a biosynthetic way for esters besides the other two sources (from raw materials and spontaneous chemical esterification), is the main route for esters synthesis [24]. Previous studies showed that ester production reactions during *baijiu* fermentation was primarily catalyzed by esterases, alcohol acyltransferases, and hemiacetal dehydrogenases [25]. Furthermore, the reported literature indicated that various microorganisms were associated with ester production, including fungi (genera *Aspergillus*, *Monascus* and *Trichosporon*), yeasts (genera *Candida*, *Pichia*, *Issatchenkia*, *Saccharomyces*, *Kazachstania*, *Zygosaccharomyces*, etc.), and bacteria (*Clostridium*, *Bacillus*, *Rummmeliibacillus,* and *Lactobacillus*) [20,24,26,27]. However, the community succession, assembly patterns, and metabolic functions of the ester-producing microbes during the production of *Nongxiangxing baijiu* remained not fully clear. 

In this research, our primary focus was on analyzing the structure of microbial communities, succession, and assembly patterns in fermentation grains through the methods of culture-independent combined with bioinformatics analysis. Then, the flavor-producing microorganisms, especially ester-producing microorganisms, were revealed. Based on this, strains with potential for ester production were screened through a culture-dependent method. The results of this study may improve our comprehension of functional microorganisms for ester production and the formation rules of ester compounds, so as to lay the groundwork for enhancing the quality of *Nongxiangxing baijiu*.

## 2. Materials and Methods

### 2.1. Fermentation and Sample Collection

The original FG were obtained from *Nongxiangxing baijiu* company in Luzhou, Sichuan Province, China. Each 400 g FG were fermented in a sealed container at 28 °C, after adjusting the moisture content to 60% with water. The samples were gathered and ground on days 0, 7, 14, 21, and 28. Then, the samples were subsequently separated into three portions and stored for physicochemical assessment (−20 °C), volatiles detection (−20 °C), and DNA extraction (−80 °C), respectively. We established three biological replicates throughout the experiment. For each test group, three parallel groups were established.

### 2.2. Measurement of the Physicochemical Properties

The properties were assessed following the technique of DB 34/T 2264-2014. All of the FG samples were measured in triplicate to ensure precision.

### 2.3. Analysis of Volatile Metabolites

The methods and equipment used for determining flavor compounds are based on approach of He et al., using methyl octanoate (73 mg/L) as internal standard [28]. Specifically, a headspace solid-phase microextraction gas chromatography–mass spectrometry (HS-SPME-GC–MS) method was used to detect the volatile compounds with the 50/30 μm DVB/CAR/PDMS fiber (Supelco, Inc., Bellefonte, PA, USA) as the extractor. After being extracted, the volatile compounds were detected by a Shimadzu (Japan) GCMS-QP2010SE system (Thermo Fisher Scientific, Waltham, MA, USA) equipped with a DB-WAX capillary column (60 m × 0.32 mm × 0.25 μm; Agilent Technologies, Santa Clara, CA, USA). The constituents were identified and selected (similarity > 80%) by comparing with the NIST05 spectrum database.

### 2.4. DNA Extraction, PCR Amplification and Illumina MiSeq Sequencing

The extraction of DNA was based on previous reports [29]. The PCR mix comprised 4 μL of 5× Fast Pfu buffer, 2 μL of 2.5 mM dNTPs, 0.8 μL of each 5 μM primer, 0.4 μL of Fast Pfu polymerase, 10 ng of template DNA, and ddH2O to 20 μL. Cycling conditions included 95 °C for 3 min, 27 cycles of 95 °C for 30 s, 55 °C for 30 s, 72 °C for 45 s, followed by a 10 min extension at 72 °C and a 4 °C hold. PCR products were purified on a 2% agarose gel, quantified with a Qubit 4.0, pooled, and sequenced using an Illumina PE300/PE250 platform by Majorbio Bio-Pharm Technology Co. Ltd. (Shanghai, China).

### 2.5. Processing of Sequencing Data

The raw FASTQ files were demultiplexed, followed by quality filtering using fastp and sequence merging with FLASH under the following conditions: sequences were truncated if the average quality score in a 50 bp sliding window fell below 20, and any reads shorter than 50 bp or containing ambiguous bases were discarded; sequences with overlapping regions exceeding 10 bp and a mismatch ratio of ≤0.2 were merged, while non-merged sequences were excluded. The samples were distinguished using barcodes and primers with exact barcode matching, allowing up to 2 mismatches for primers [30,31]. Filtered sequences were clustered into OTUs at 97% similarity using UPARSE, with chloroplast sequences removed. To account for sequencing-depth differences, 16S rRNA and ITS gene sequences were rarefied to 40,770 and 36,432 sequences per sample [32,33]. OTU taxonomy was assigned using the RDP Classifier and the Silva database [34]. Metagenomic functional predictions were carried out using PICRUSt2, following its established protocol [35].

### 2.6. Screening for Ester Producing Yeasts

The formulation for the esterification screening (ES) medium consists of 0.5% peptone, 0.3% yeast extract, 0.1% tributyrin, and 2% agar powder. A 5 g sample of FG was dissolved in 95 mL of sterilized water and mixed thoroughly. After appropriate dilution, the suspensions of different concentrations were transferred to ES plates and incubated at 28 °C. Colonies with various morphologies were then isolated and examined microscopically. Selected single colonies were streaked onto fresh medium and cultured at 30 °C, with streaking repeated until isolated colonies were obtained. After screening the strains based on the morphology, the molecular identification was carried out for 17 yeast strains by sequence analysis of 26S rDNA.

### 2.7. Statistical Analysis and Visualization

To evaluate the statistical significance of the physicochemical properties, a one-way analysis of variance (ANOVA) was employed, with significance determined at a threshold of *p* < 0.05. A correlation network analysis was carried out utilizing the Spearman correlation coefficient and achieved through Gephi (version 0.9.2). RDA was plotted using R vegan. The Pearson correlation between microbiota and environmental parameters was examined using a Mantel test. This analysis was conducted in R (version 4.2.0). PICRUSt2 was utilized to evaluate the functional profile of microbial community in the FG, while pathway enrichment of metabolites was collected based on the Kyoto Encyclopedia of Genes and Genomes (KEGG) database [35].

## 3. Results and Discussion

### 3.1. Variations of Physicochemical Properties of FG

In this study, the physicochemical properties of FG, including moisture content, alcohol content, titratable acidity, reducing-sugar content, and starch content, were investigated, which characterized the fermentation process (Figure S1). The moisture content increased rapidly from 59.72% to 65.55% on days 0 to 28. Meanwhile, the titratable acidity showed an upward tendency during the whole fermentation process, with 1.76 (mL/g FG) as minimum value and 4.70 (mL/g FG) as maximum value on day 0 and day 28, respectively (Figure S1A); analogous results were also observed by Xu et al. [36]. The changes in the content of reducing-sugar and starch were opposite to that of titratable acidity, which displayed a consistent downward trend until the completion. The alcohol content fluctuated up and down during the fermentation phase, which increased first, decreased later, and displayed an upward trend in the end, with a maximum value of 4.67% (*v*/*v*) on day 14 (Figure S1B). Hu et al. [19] also recorded analogous results. As the fermentation proceeded, the consumption and utilization of starch and reducing-sugars occurred due to the collaborative actions of various microorganisms and enzymes and ethanol and water were produced during this period. Consequently, the fermentation process was indicated by the utilization of starch and reducing-sugars, along with the accretion of acids and ethanol [26].

### 3.2. Profiles of Volatile Metabolites in FG

The FG samples were treated, and volatiles were detected to track and assess the variations in volatile substances, especially esters, throughout fermentation process. A total of 58 volatile flavors were detected, consisting of 33 esters, 11 acids, 7 alcohols, 3 ketones, 3 aldehydes, and 1 phenol (Table S1). In this work, esters constituted the majority of the volatile flavor compounds identified, while acids and alcohols were present in smaller proportions, with substances analogous to those reported literature [37,38]. A previous work showed that the fermentation grains were the immediate origin of volatile substances in *Nongxiangxing baijiu* owing to their similar diversity of compounds [36].

**Figure 1:**
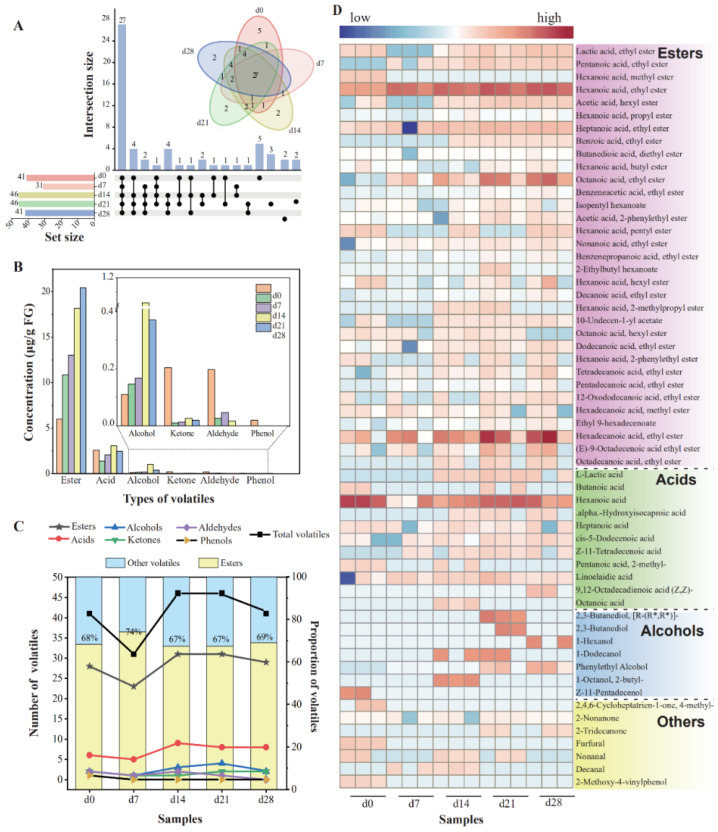
Changes in the contents and numbers of the volatile compounds from FG samples during fermentation. (**A**) The number of shared and unique volatiles presented by Upset diagram; (**B**) concentration of volatiles; (**C**) numbers and proportion of volatiles of FG; (**D**) heatmap of 58 volatiles in FG during fermentation.

During the fermentation, the number of volatile compounds dropped from 41 (day 0) to 31 (day 7), then rose to 46 (day 14 and day 21), which finally declined to 41 (Figure 1A). Meanwhile, the number of esters decreased from 28 (day 0) to 23 (day 7), then increased to 31 (days 14–21), and became 29 in the end. The content and proportion of various esters greatly affected *baijiu*’s flavor profile [24]. Esters constituted the predominant volatiles in all the FG samples, making up 64% to 88% of the total volatiles., with ethyl hexanoate accumulating the fastest and most (11.32 μg/g FG in the end). The changes in amount and content of esters were positively correlated with those of the total volatile components (Figure 1B,C). Furthermore, the variations in the content of volatiles are displayed in Figure 1D. With the extension of the fermentation time, a majority of volatiles displayed an upward trend, including most esters, some acids, phenylethyl alcohol, and 2-nonanone. A total of 11 volatiles became higher first and then showed a general decreasing trend. Eighteen volatiles began to generate during days 0–7. Among them, 83.33% of them were esters, which were all ethyl esters. This may be related to the abundance of ethanol and enzymes that catalyze the generation of ethyl esters [25].

### 3.3. Structure and Dynamics of Microbial Community

The microbial community structures of FG and the dynamic characteristics were explored by applying Illumina HiSeq sequencing. After the quality control, 738,662 effective sequences from a V3-V4 region and 954,866 effective sequences from an internal transcribed spacer region were collected from 15 FG samples. The amounts of high-quality sequences varied from 40,770 to 72,343 and 36,432 to 91,736 for each sample, respectively. The ratio of high-quality sequences was 99.07% and 99.87% (Table S2). All FG samples showed coverage values greater than 99%, and the rarefaction curve, calculated from the OTU count, reached the saturation threshold, suggesting that the sequences were appropriate to reflect the community structure (Figure S2).

**Figure 2:**
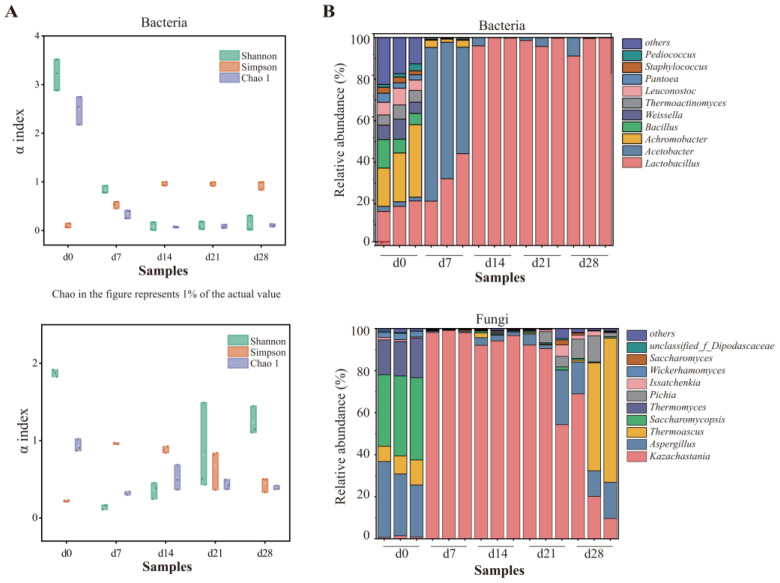
Changes in bacterial and fungal communities during fermentation: (**A**) microbial diversity; (**B**) distribution of the microbial community at genus level.

Firstly, the diversity and richness of the microbial communities were assessed using standardized sequences. In terms of bacteria, the Shannon index decreased to the minimum on the day 14, as shown in Figure 2A, suggesting that the bacterial diversity declined sharply and then gradually stabilized. A Chao1 index showed that the bacterial richness declined to its lowest point (day 14) and then exhibited a slight increase. As for fungi, the Shannon index exhibited a trend comparable to that observed in bacteria, with minimum on the day 7. However, the Chao1 index fluctuated slightly as fermentation progressed, while decreased significantly from day 0 to day 7. Generally, changes in environmental conditions, like higher ethanol content, moisture content, and acidity during fermentation, may lead to the elimination of microorganisms that were not fit for the habitat, causing a massive decrease in microbial diversity within 7 days. Comparable findings were also noted by Mu et al. [27]. Subsequently, the diversity of bacteria rebounded slightly, while fungal microbial communities increased. 

The analysis of the microbial community demonstrated that 23 phyla, including 16 bacterial and 7 fungal phyla, were identified. The most dominant bacterial phylum was Firmicutes, showing a relative abundance of greater than 96.83% at the end of fermentation and 77.19% in average, followed by Proteobacteria and Actinobacteria. Ascomycota was the most prevalent fungal phylum, accounting for more than 99% (Figure S3). At the genus level, 350 genera (251 of bacteria and 99 fungi) were detected in total. On the day 0 and day 7, the most dominant bacterial genera were *Achromobacter* and *Acetobacter*, with the relative abundance of 26.18% and 63.52%, respectively. With the extension of fermentation time, an obvious increase in abundance of *Lactobacillus* was noted (from 32.53% on day 7 to 98.52% on day 28). *Lactobacillus*, following the most dominant bacterial genus on the day 0 and day 7, remained dominant from day 14 through the completion of fermentation, accounting for 91.06–99.80% in each sample collected from day 28 (Figure 2B). However, the comparative quantity of *Lactobacillus* improved, while the acidity accumulated continuously. *Lactobacillus* trended to be preeminent in abundance in late phases, which was described as a biomarker throughout *Nongxiangxing baijiu* fermentation [21,39]. It was acknowledged for its capacity to transform carbohydrates into lactic acid, an important precursor of esters [40].

In terms of fungi, *Saccharomycopsis* (37.05%), *Aspergillus* (30.09%), and *Thermomyces* (17.30%) were dominant in the top 10 genera in samples collected from day 0, accounting for over 80% of the microbial composition. In the samples collected from day 7, day 14, and day 21, *Kazachastania* was the most prominent fungal genus, with comparative abundance exceeding 90%. At the completion of fermentation, *Kazachstania*, *Thermoascus,* and *Aspergillus* were detected to be dominant fungal genera. This finding was corresponding to earlier studies [26,40]. *Kazachstania* was also identified as the key functional genus during the fermentation of *Nongxiangxing baijiu*, contributing to the synthesis of ethanol and flavor alcohols [27,36,41].

### 3.4. Microbiota Contributing to Ester Formation

As described in previous study, microorganisms were pivotal in the synthesis of volatiles [24]. To clarify the association between the microorganisms and volatiles, especially esters, a Spearman’s correlation analysis between 31 volatiles and top 20 genera (dominant 10 fungal and top 10 bacterial genera) was performed (Figure S4). As shown in Figure S5, *Lactobacillus* displayed positive association with almost all of the abundant esters. *Acetobacter* exhibited positive associations with hexanoic acid, ethyl ester, hexadecanoic acid, ethyl ester, octadecenoic acid, ethyl ester, hexanoic acid, butyl ester and tetradecanoic acid, ethyl ester. While eight bacterial genera (*Achromobacter*, *Bacillus*, *Weissella*, *Thermoactinomyces*, *Leuconostoc*, *Pantoea*, *Staphylococcus,* and *Pediococcus*) were correlated negatively with most esters, acids and alcohols. The possible reason was that *Lactobacillus* and *Acetobacter* could produce the precursor of esters (lactic acid and acetic acid), which was supported by previous studies, suggesting that they were strongly linked to the acidity or the generation of acids [42,43,44]. As for fungi, there were more genera showed a positive association with volatile compounds. *Pichia* showed strong positive correlation (r greater than 0.7) with 11 esters, cis-5-Dodecenoic acid and phenylethyl alcohol. *Issatchenkia* and *Saccharomyces* positively correlated with most of the abundant esters. 

**Figure 3:**
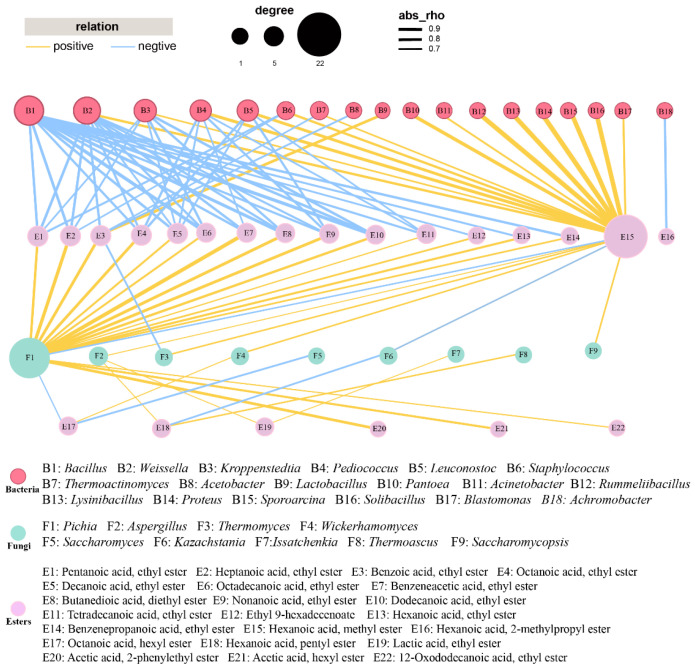
Correlation between 33 esters and dominant genera (relative abundance > 1%) in FG samples. Yellow and blue edges represent positive and negative correlations, respectively. For networks of bacteria and fungi, Spearman’s r > 0.7 or < −0.7 and Spearman’s r > 0.6 or < −0.6, respectively, *p* < 0.05.

To examine the interactions between the key genera and esters, a network analysis was carried out (Figure 3). For the correlation network, most esters displayed an inverse correlation with bacteria and positive association with fungi. Fourteen out of the eighteen bacteria showed a positive association with methyl hexanoate. One effective connection edge of Lactobacillus was observed in Figure 3, indicating that Lactobacillus were significantly showing a positive association with ethyl benzoate. *Bacillus*, *Weissella*, *Kroppenstedtia,* and *Pediococcus* showed negative relationships with at least 5 esters. As for fungi, *Pichia* showed strong positive correlation with 17 esters, while comparable findings were observed by previous research [27,45]. *Aspergillus* and *Issatchenkia* displayed positive correlations with ethyl lactate. *Aspergillus*, *Thermomyces*, *Wickerhamomyces,* and *Saccharomycopsis* were positively correlated with methyl hexanoate. *Thermoascus* showed a significant positive association with pentyl hexanoate.

Previous research indicated that Pichia might be the contributor to esterase activity, showing positive linkages with many esters, including ethyl hexanoate and ethyl pentanoate [27,46,47]. *Aspergillus* was found to function as the producer of both saccharifying agents and flavor substances [48]. *Thermomyces* was also reported as dominant fungal genus for many times. However, its function in *baijiu* fermentation was rarely investigated [49]. *Wickerhamomyces* was used to regulate the generation of flavor compounds, which changed the content of ethyl acetate as well as fluctuations in other flavor compounds [50,51]. *Issatchenkia* was observed to have the capacity of producing various esters during *Nongxiangxing baijiu* fermentation and showed the potential in enhancing the quality of *baijiu* [26,46]. Similarly, *Saccharomycopsis* was used for regulating the microbiota in daqu in order to improve the flavor compounds profile [52].

As reported, ester formation was mainly catalyzed by esterases, alcohol acyltransferases, and hemiacetal dehydrogenases, suggesting that increasing the concentrations of acid, alcohol, acyl-CoA, or other precursors of esters formation is one of the strategies to increase ester yield [25]. Some esters were correlated with multiple microorganisms, indicating that the generation of esters may rely on the synergistic influence of multiple microorganisms. 

### 3.5. Microbiota Assembly in FG during the Brewing of Baijiu

**Figure 4:**
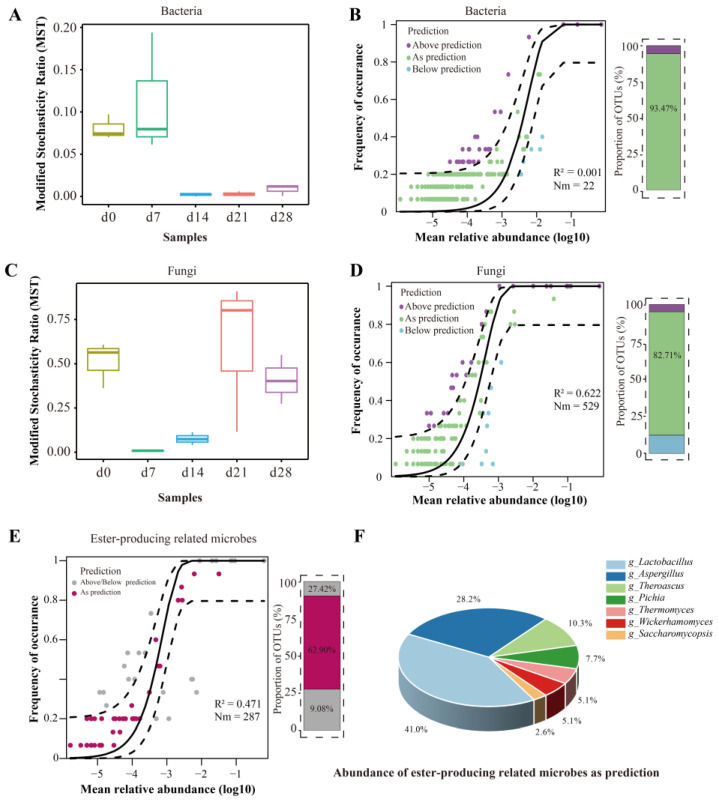
Microbiota assembly patterns in FG during fermentation. (**A**–**D**) Modified stochasticity ratio (MST) and Sloan neutral model of bacteria and fungi; (**E**) Sloan neutral model of ester-producing related microbes; (**F**) abundance of ester-producing related microbes as prediction.

In order to investigate the microbiota assembly, the modified stochasticity ratio (MST) index and the Sloan neutral model were employed to evaluate the roles of deterministic and stochastic processes to the assembly of *Nongxiangxing baijiu* microbiota (Figure 4). On a Bray–Curtis dissimilarity index, the MST dependence showed that bacteria were primarily controlled by deterministic processes (MST < 0.5), with stronger impact of stochastic processes observed during the latter part of fermentation (Figure 4A). As shown in Figure 4B, the Sloan neutral model hardly explained the succession of bacterial communities, with an R^2^ value of 0.001, indicating that deterministic processes shaped the microbial composition again. These results might be attributed to the buildup of selective pressures resulting from shifts in ecological factors, making the rapid growth of microorganisms with higher adaptability and resulting in homogeneous selection [53]. The selection processes retained microorganisms with exceptional adaptability and enhanced functions of particular communities [54]. The reduction in the reducing-sugar content and starch content might potentially introduce more stochastic processes in bacterial community as fermentation progresses. Meanwhile, the fast succession rate of other microorganisms like Lactobacillus might be a reason for its deterministic processes [27].

**Figure 5:**
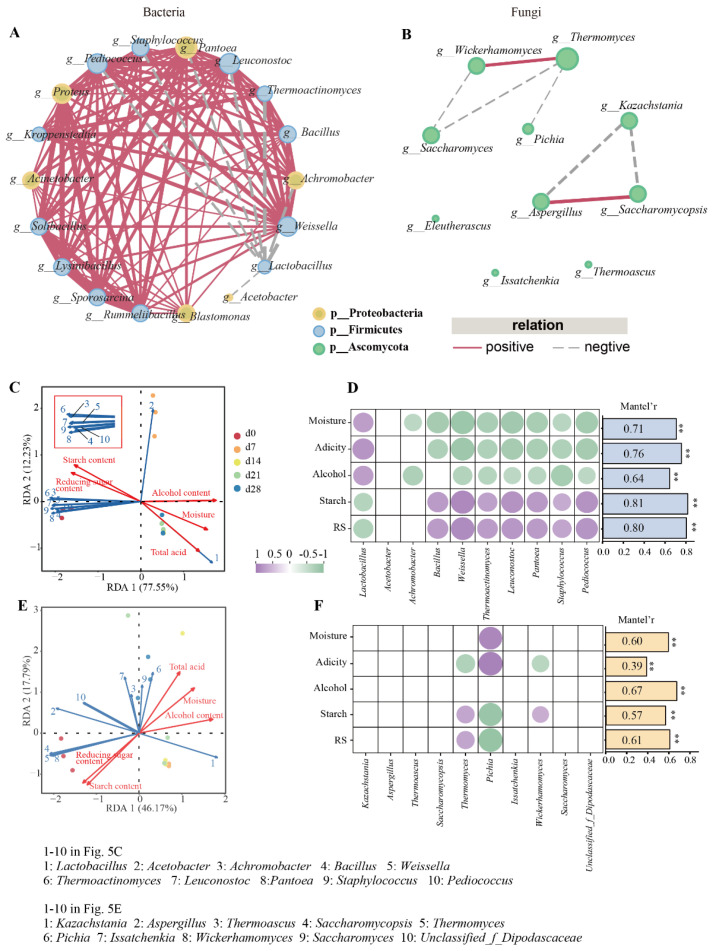
Interactions among microbes and their correlations with physicochemical properties: (**A**,**B**) co-occurrence networks based on spearman’s correlation coefficients of bacterial (**A**) and fungal genera (**B**); (**C**,**D**) correlation of bacteria and physicochemical factors based on redundancy analysis and Mantel test, respectively; (**E**,**F**) correlation of fungi and physicochemical factors based on redundancy analysis and Mantel test, respectively. Nodes represent individual genus. Red and gray edges represent positive and negative correlations, respectively. For interaction network, Spearman’s r > 0.7 or < −0.7, *p* < 0.01. The color of the node indicates the classification of the genus at the phylum level. Correlations with Spearman’s r > 0.7 or < −0.7, *p* < 0.05 are shown in the heatmap. ** *p* < 0.01.

As for the fungal community, the assembly pattern was more frequently governed by stochastic processes. However, the deterministic process also exhibited a non-negligible proportion that must be considered, especially on day 7 and day 14 (Figure 4C). Using the Sloan neutral model, it was evaluated that approximately 50% of the associations between the frequencies at which fungal OTUs occur and their changes in abundance could be explained. This model clarified 62.2% of the overall variance observed in the community throughout the entire fermentation period (Figure 4D). The percentage of OTUs exhibiting a neutral distribution was 82.71%. The outcome in agreement with an earlier study [55]. Notably, the results of Spearman’s correlation and the Mantel test in Figure 5 also showed higher effect of environmental selection on bacterial succession than that of fungi. It was indicated that fungal microbes might have a higher tolerance to acidity or biotic interaction selection.

In order to determine the assembly processes of the ester-producing-related microbes, the Sloan neutral model was employed (Figure 4E). As shown in Figure 4E, about 47.1% of the variation in community being elucidated during the whole fermentation with 62.90% of OTUs neutrally distributed. For the OTUs as prediction, most of them were Lactobacillus and Aspergillus (Figure 4F).

### 3.6. Interactions among Microbes and Their Correlations with Physicochemical Properties

As the fermentation continued, the succession of microbial community led to the variations in FG, which affected microbiota dynamics at the same time. The interaction between microbial community and physicochemical properties were strongly associated with the formation of flavor substances. In this study, the correlations among microbiota and correlations with physicochemical properties were revealed (Figure 5).

The interactions among genera (abundance greater than 1%) in FG samples were investigated through co-occurrence network analysis built upon Spearman’s correlation (|r| > 0.7, *p* < 0.05). A sum of 111 and 7 edges were observed in bacterial and fungal network, respectively, suggesting that stronger interactions among bacterial communities existed. *Lactobacillus* showed a negative correlation with eight bacteria (Figure 5A). The remaining bacterial genera, except for *Acetobacter*, mutually showed positive correlation. *Aspergillus* exhibited positive associations with *Saccharomycopsis* and *Kazachstania*. *Thermomyces* exhibited a highly positive relationship with *Wickerhamomyces* and showed negative correlation with *Pichia* and Saccharomyces. As shown in Figure 5A, Proteobacteria, Firmicutes, and Ascomycota were abundant phyla, accounting for 66.67%, 33.33%, and 100.00% of total nodes in bacterial and fungal network, respectively. Moreover, the proportion of positive links were 92.79% and 28.57% for fungi and bacteria, respectively.

The relationship between physicochemical properties and microbiota were further assessed based on both the redundancy analysis (RDA) and Mantel test (Figure 5). According to RDA results (Figure 5C,E), the bacterial and fungal models explained 89.78% and 60.96% of the total variance among samples, respectively. Starch and reducing-sugar were associated with the community from sample d0, while moisture, acidity, and alcohol were associated with the bacterial community collected from days 14–28 and fungal community collected from days 7–21. These results suggested that correlations between bacterial communities and physicochemical parameters were stronger than those of fungi and this finding agreed with previous findings [27]. RDA results (Figure 5C) also showed the positive correlation between acidity and Lactobacillus. These indicated that Lactobacillus might be important acid-producing bacteria, and comparable findings were also documented by Guan et al. [26]. 

Additionally, Mantel tests and correlation analysis between microbial communities and environmental factors demonstrated that bacterial communities were affected by environmental properties, while only *Thermomyces*, *Pichia,* and *Wickerhamomyces* were significantly related to environmental factors (Figure 5D,F). More specially, it was shown that *Lactobacillus* showed positive associated with total acid, moisture, and alcohol content. Other seven genera, namely *Bacillus*, *Weissella*, *Thermoactinomyces*, *Leuconostoc*, *Pantoea*, *Staphylococcus,* and *Pediococcus*, displayed positive correlations with starch and reducing-sugar contents. Notably, ethanol had almost no clearly noticeable impact on fungal communities.

### 3.7. Isolation and Identification of Ester-Producing Microbes from FG

To further validate the capacity of ester-producing microbes, a total of 17 yeast strains were selected from FG using an ester-producing screening medium. According to morphological characteristics and phylogenetic analysis, 10 strains were identified as *Saturnispora* sp. (FG3, FG 20, FG22, FG24, and FG26) and *Saturnispora silvae* (FG31, FG32, FG41, FG42, and FG43), while the other 7 strains were identified as *Zygosaccharomyces bailii* (FG1, FG5, FG6, FGP12, FG17, and FG18) and *Zygosaccharomyces* sp. (FG19), with an identity higher than 99%, except for FG24 (Table 1). However, neither of these two fungal genera was detected through ITS sequencing. This might suggest that both types of fungi genera were more appropriate for growth on the culture medium than the other microorganisms detected.

*Zygosaccharomyces bailii* was widely reported in *Nongxiangxing baijiu* and was also proved to be a dominant species in sauce flavor *baijiu* [56]. It demonstrated strong tolerance to various stresses, and it was regarded as a potential producer of various ethanol and aromatic compounds in *baijiu* fermentation, including isoamylol, pentanol, and isobutanol [57]. However, *Saturnispora silvae* was rarely reported in *baijiu* fermentation. Yan et al. [58,59]. reported that *Saturnispora silvae* was detected in pit mud and daqu using denaturing gradient gel electrophoresis (DGGE) approach, and it had a positive connection to levels of hexanoic acid based on canonical correspondence analysis (CCA), which might be associated with its ester-producing ability. Nevertheless, future studies will be essential to fully understand the characteristics and role in *baijiu* fermentation.

**Figure 6:**
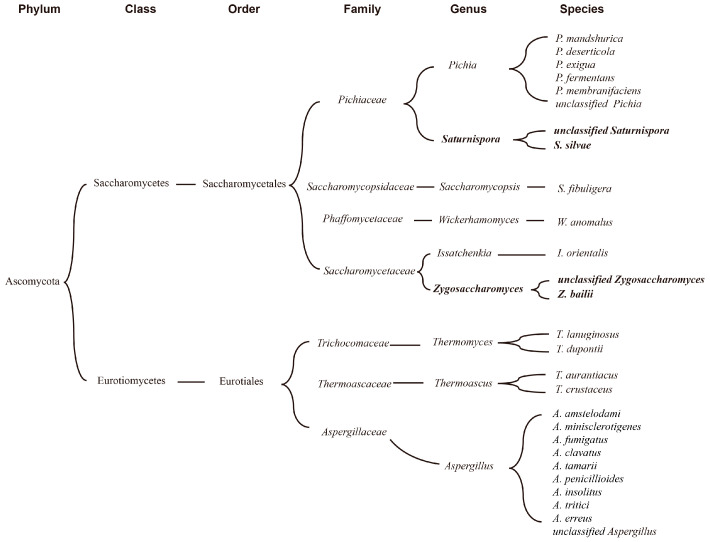
Summary of microbes related to ester production obtained via correlation analysis and culture-based method.

According to the results of correlation analysis, 7 fungal genera related to ester production, namely *Pichia*, *Aspergillus*, *Wickerhamomyces*, *Thermomyces*, *Saccharomycopsis*, *Issatchenkia,* and *Thermoascus*, were selected from the FG samples. To further understand the ester-producing related microbes in FG samples, a summary of microbes related to ester production obtained via correlation analysis and a culture-based method was made. As shown in Figure 6, they could be divided into two classes, namely Saccharomycetes and Eurotiomycetes. Six out of nine belong to the order Saccharomycetales, and others belong to Eurotiales. Saturnispora and Pichia belong to the Pichiaceae family, while *Zygosaccharomyces* and *Issatchenkia* belong to the Saccharomycetaceae family.

### 3.8. Prediction of the Microbial Functions in Fermented Grains

**Figure 7:**
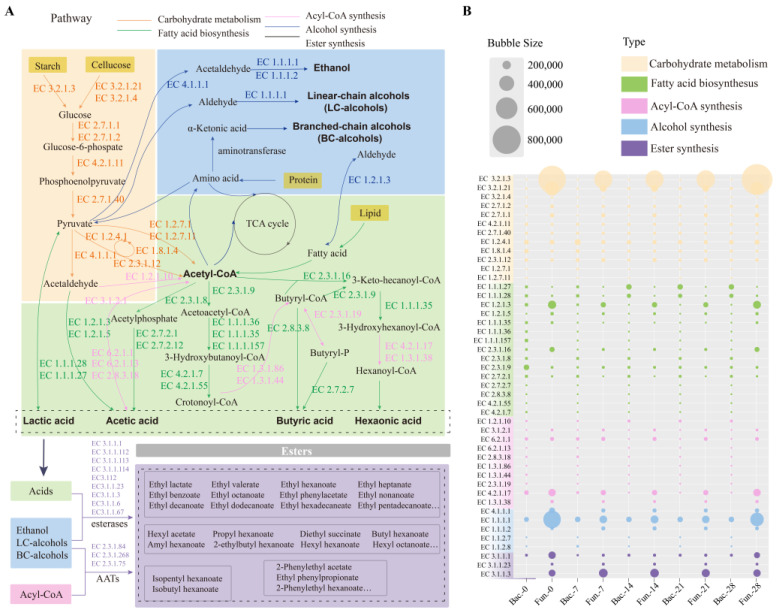
Prediction of the microbial functions in synthesis of esters in FG: (**A**) metabolic pathways diagram related to esters formation in FG fermentation; (**B**) the relative abundance of the main enzymes involved in the metabolic pathways.

Using sequencing data, the functional characteristics of microbiota in FG were predicted by PICRUSt2 (Figure 7). The enzymes and related metabolisms covering the metabolism of carbohydrates, fatty acid biosynthesis, acyl-CoA synthesis, alcohol synthesis, and ester synthesis were displayed, which were mainly involved in carbohydrate degradation and flavor compounds formation in FG samples. It was reported that two pathways of microbial synthesis of esters were identified as the primary for synthesizing esters in *baijiu* fermentation [24,25]. Generally, acids and alcohols were catalyzed to produce esters, and the frequently reported enzymes were mainly esterase carboxylesterase (EC 3.1.1.1) and lipase (EC 3.1.1.3). Another pathway was catalyzed by alcohol acyltransferases (AATs), which was also the main synthetic route for ethyl acetate.

In this study, 45 enzymes were selected and classified into five categories, and the annotations of these enzymes were displayed in Table S4. A total of twelve enzymes were annotated in carbohydrate metabolism (such as EC 3.2.1.3, EC 3.2.1.21, EC 3.2.1.4, and EC 2.7.1.2). Most of the enzymes were involved in glucose metabolism, except for enzymes EC 4.2.1.11, EC 1.8.1.4, EC1.2.7.1, and EC 1.2.7.11, which showed a higher abundance in fungi than that in bacteria. Similar results were also found in enzymes engaged in ethanol metabolism (EC 4.1.1.1, EC 1.1.1.1 and EC 1.1.1.2), which was a result of the abundance of filamentous fungi [60]. As important precursors of esters formation, fatty acids were generated from multiple pathways. In this study, the formation of the four major organic acids and their corresponding ethyl esters were focused. As shown in Figure 7, enzymes responsible for the metabolism of the four major acids were clearly observed. During FG fermentation, hexanoic acid was important precursor for ethyl caproate through chain elongation pathway and esterification [22,61]. Overall, aldehyde dehydrogenase (EC 1.2.1.3), acetyl-CoA C-acetyltransferase (EC 2.3.1.9, EC 2.3.1.16), and enoyl-CoA hydratase (EC 4.2.1.17), which were related to metabolism of hexanoic acid, were significantly highly enriched in samples. This corresponded to the high content of ethyl caproate (Figure 1D). Thus, it is crucial to investigate the microorganisms with capability to regulate the production of esters and their precursor substances for improving the quality of *baijiu*. Notably, 14 enzymes related to four major esters synthesis were only produced by bacteria, proving the importance of bacteria in four major ethyl esters metabolism [62]. Meanwhile, Trans-2-enoyl-CoA reductase (EC 1.3.1.38) only secreted by fungal communities. This demonstrated that both bacteria and fungi were involved in the synthetic pathways of esters. More than nine and three enzymes could catalyze the synthesis of esters (Figure 7A) in *baijiu* through esterification and alcohol acyl-transfer reaction, respectively (data based on KEGG database). However, three enzymes were predicted in the samples in this study, while carboxylesterase (EC 3.1.1.1) was shared by bacterial and fungal microorganisms, and acylglycerol lipase (EC 3.1.1.3) and triacylglycerol lipase (EC 3.1.1.23) were only secreted by fungal communities. Acylglycerol lipase presented higher abundance compared to the other two enzymes (Figure 7B), indicating that it may be an important enzyme for catalyzing the formation of esters in the brewing of *Nongxiangxing baijiu*.

## 4. Conclusions

In summary, this work investigated the community succession, assembly patterns, and function of ester-producing microbes in FG samples during the production of *Nongxiangxing baijiu*. The results suggested that the bacterial genus *Lactobacillus* and the fungal genera *Pichia*, *Aspergillus*, *Wickerhamomyces*, *Thermomyces*, *Saccharomycopsis*, *Issatchenkia,* and *Thermoascus* were significantly related to the generation of esters based on correlation analysis. The microbial assembly analysis showed that bacteria in FG were predominantly governed by deterministic processes, while the assembly of fungi was more frequently shaped by stochastic process. The results of the correlation analysis indicated that bacteria in FG were more sensitive to environmental factors. Then, *Saturnispora silvae* and *Zygosaccharomyces bailii* with capacity of producing esters were screened via culture-based methods. To our knowledge, this is the first instance that *Saturnispora* has been screened and isolated from FG. In addition, the microbial functions in ester synthesis in FG were estimated according to PICRUSt2 analysis, and the findings suggested that fungi in FG had a higher potential for synthesizing ethanol, while bacteria had a higher potential for synthesizing acyl-CoA. The synthesis of esters in the FG samples was mainly catalyzed by esterase rather than alcohol acyltransferases. These findings may provide information about the related ester-producing microbes in *Nongxiangxing baijiu* fermentation, which will facilitate the effective management of *baijiu* fermentation through the application of functional microorganism.

## Figures and Tables

**Table 1:** Identification of strains isolated from fermentation grains.

Strains	Closest Relative (NCBI Accession No.)	Identity (%)
FG3	*Saturnispora* sp. YPM2 (MG649461.1)	99.10
FG20	*Saturnispora* sp. YPM2 (MG649461.1)	100.00
FG22	*Saturnispora* sp. YPM2 (MG649461.1)	100.00
FG24	*Saturnispora* sp. LCF-02 GY16S01 (HM461664.1)	97.73
FG26	*Saturnispora* sp. YPM2 (MG649461.1)	99.09
FG1	*Zygosaccharomyces bailii* SPF204 (KY653135.1)	99.82
FG5	*Zygosaccharomyces bailii* ML3 (KY296086.1)	99.66
FG6	*Zygosaccharomyces bailii* NT-110 (MN371952.1)	99.83
FG12	*Zygosaccharomyces bailii* ML3 (KY296086.1)	99.66
FG17	*Zygosaccharomyces bailii* J13 (PP033914.1)	99.65
FG18	*Zygosaccharomyces bailii* J13 (PP033914.1)	99.65
FG19	*Zygosaccharomyces* sp. H2Y32 (JF781363.1)	99.82
FG31	*Saturnispora silvae* F610 (MK110170.1)	100.00
FG32	*Saturnispora silvae* LS 151 (OR622484.1)	100.00
FG41	*Saturnispora silvae* FN9S03 (EF460523.1)	100.00
FG42	*Saturnispora silvae* LS 121 (OR622482.1)	100.00
FG43	*Saturnispora silvae* F610 (MK110170.1)	100.00

## Data Availability

The data from this study can be obtained upon reasonable request. For additional information, please contact the corresponding author.

## Supplementary Materials

The following supporting information can be downloaded at https://www.mdpi.com/article/10.3390/foods13193169/s1: Figure S1: The physicochemical properties of FG samples. (A) moisture and acidity, (B) alcohol content, starch content and reducing sugar content of FG samples. Each datum is the average of three measurements. Error bars indicate standard deviations (n = 3); Figure S2: Rarefaction curve of (A) bacteria and (B) fungi of FG samples; Figure S3: Relative abundance of (A) bacteria and (B) fungi at the phylum level; Figure S4: Spearman’s correlation between microbial communities and flavor compounds. The analyzed elements include the top 20 genera, the top 20 esters, and some acids and alcohols with significant variation; Table S1: Volatile compounds detected in fermentation grains during fermentation; Table S2: High quality sequence based on 16S rRNA and ITS sequencing; Table S3: The microbial community richness and diversity indices for microbial communities based on 16SrRNA and ITS sequencing; Table S4: The enzymes related to ester formation of microbial community in FGs predicted by PICRUSt2 and their annotation.
