# Supplementary material for: Exploring Community Succession, Assembly Patterns, and Metabolic Functions of Ester-Producing-Related Microbiota during the Production of Nongxiangxing baijiu
Source: Foods. 2024 Oct 5;13(19):3169. doi: 10.3390/foods13193169 (PMC11476053; doi:10.3390/foods13193169)
Supplement: Supplementary file 1 [file foods-13-03169-s001.zip › foods-3202250-supplementary.pdf]

Figure S1. The physicochemical properties of FG samples. (A) moisture and acidity, (B) alcohol content, starch content and reducing sugar content of FG samples. Each data is the average of three measurements. Error bars indicate standard deviations (n=3).

Figure S2. Rarefaction curve of (A) bacteria and (B) fungi of FG samples.

Figure S3. Relative abundance of (A) bacteria and (B) fungi at the phylum level.

Figure S4. Spearman's correlation between microbial communities and flavor compounds. The analyzed elements include the top 20 genera, the top 20 esters, and some acids and alcohols with significant variation.

Table S1. Volatile compounds detected in fermentation grains during fermentation.

	Volatile compounds	Retention time (min)	Concentration (μg/g FG)				
			d0	d7	d14	d21	d28
**Esters (33)**	Lactic acid, ethyl ester	5.785	0.172 ± 0.065		0.061 ± 0.053	0.256 ± 0.176	0.427 ± 0.118
	Pentanoic acid, ethyl ester	8.765		0.039 ± 0.00	0.082 ± 0.057	0.152 ± 0.108	0.172±0.042
	Hexanoic acid, methyl ester	10.148	0.111 ± 0.031				
	Hexanoic acid, ethyl ester	13.034	2.102 ± 0.122	6.466 ± 2.06	7.354 ± 1.724	9.753 ± 3.441	10.777 ± 3.041
	Acetic acid, hexyl ester	13.505	0.025 ± 0.033		0.034 ± 0.003	0.178 ± 0.037	0.176 ± 0.05
	Hexanoic acid, propyl ester	16.408	0.027 ± 0.008	0.019 ± 0.004	0.051 ± 0.026	0.038 ± 0.001	0.04 ± 0.018
	Heptanoic acid, ethyl ester	16.518	0.304 ± 0.089	0.592 ± 0.048	0.743 ± 0.165	0.969 ± 0.276	1.023 ± 0.111
	Benzoic acid, ethyl ester	18.810			0.007 ± 0.001	0.007 ± 0.001	0.01 ± 0.002
	Butanedioic acid, diethyl ester	19.247	0.007 ± 0.001	0.006 ± 0.001	0.011 ± 0.001	0.012 ± 0.001	0.013 ± 0.004
	Hexanoic acid, butyl ester	19.597	0.335 ± 0.108	0.234 ± 0.075	0.174 ± 0.075	0.344 ± 0.044	0.358 ± 0.083
	Octanoic acid, ethyl ester	19.784	0.471 ± 0.123	0.815 ± 0.166	0.966 ± 0.23	1.292 ± 0.397	1.437 ± 0.297
	Benzeneacetic acid, ethyl ester	21.105	0.041 ± 0.008	0.044 ± 0.005	0.056 ± 0.007	0.084 ± 0.023	0.108 ± 0.029
	Isopentyl hexanoate	21.275	0.134 ± 0.04	0.114 ± 0.084	0.122 ± 0.085	0.238 ± 0.075	0.171 ± 0.117
	Acetic acid, 2-phenylethyl ester	21.571	0.016 ± 0.003	0.021 ± 0.004	0.034 ± 0.017	0.103 ± 0.077	0.093 ± 0.049
	Hexanoic acid, pentyl ester	22.570	0.109 ± 0.054		0.043 ± 0.005		0.077 ± 0.005
	Nonanoic acid, ethyl ester	22.725	0.034 ± 0.003	0.048 ± 0.012	0.059 ± 0.014	0.08 ± 0.027	0.095 ± 0.03
	Benzenepropanoic acid, ethyl ester	24.210	0.035 ± 0.011	0.052 ± 0.015	0.064 ± 0.014	0.087 ± 0.038	0.106 ± 0.046
	2-Ethylbutyl hexanoate	24.610				0.041 ± 0.008	
	Hexanoic acid, hexyl ester	25.382	0.702 ± 0.185	0.399 ± 0.075	0.472 ± 0.120	0.530 ± 0.170	0.700 ± 0.432
	Decanoic acid, ethyl ester	25.608	0.065 ± 0.018	0.077 ± 0.019	0.103 ± 0.018	0.139 ± 0.058	0.183 ± 0.072
	Hexanoic acid, 2-methylpropyl ester	27.623			0.015 ± 0.002	0.056 ± 0.035	
	10-Undecen-1-yl acetate	27.638	0.019 ± 0.024		0.021 ± 0.006	0.025 ± 0.005	0.027 ± 0.012
	Octanoic acid, hexyl ester	30.487	0.021 ± 0.013	0.008 ± 0.004	0.019 ± 0.02	0.011 ± 0.006	
	Dodecanoic acid, ethyl ester	30.793	0.024 ± 0.015	0.051 ± 0.023	0.081 ± 0.011	0.096 ± 0.05	0.136 ± 0.064
	Hexanoic acid, 2-phenylethyl ester	32.033	0.011 ± 0.004		0.016 ± 0.008	0.021 ± 0.004	0.017 ± 0.006

	Tetradecanoic acid, ethyl ester	35.380	0.071 ± 0.051	0.204 ± 0.09	0.262 ± 0.046	0.294 ± 0.167	0.397 ± 0.229
	Pentadecanoic acid, ethyl ester	36.836	0.011 ± 0.004	0.019 ± 0.01	0.025 ± 0.005	0.030 ± 0.021	0.039 ± 0.026
	12-Oxododecanoic acid, ethyl ester	37.005	0.005 ± 0.001	0.008 ± 0.005	0.01 ± 0.008	0.017 ± 0.013	0.027 ± 0.018
	Hexadecanoic acid, methyl ester	38.312	0.047 ± 0.03	0.052 ± 0.05	0.033 ± 0.029	0.039 ± 0.043	0.033 ± 0.026
	Ethyl 9-hexadecenoate	39.157	0.032 ± 0.016	0.045 ± 0.027	0.064 ± 0.018	0.099 ± 0.057	0.120 ± 0.071
	Hexadecanoic acid, ethyl ester	39.769	0.836 ± 0.404	1.412 ± 0.788	1.656 ± 0.395	2.422 ± 1.641	2.819 ± 1.896
	(E)-9-Octadecenoic acid ethyl ester	43.432	0.255 ± 0.191	0.374 ± 0.238	0.415 ± 0.122	0.755 ± 0.567	0.862 ± 0.629
	Octadecanoic acid, ethyl ester	44.111			0.013 ± 0.009	0.044 ± 0.019	0.031 ± 0.024
**Σ(Type)**			28	23	31	31	29
**Σ(Concentration)**			6.021	11.099	13.065	18.209	20.477
**Acids (11)**	L-Lactic acid	5.824			0.079 ± 0.012	0.269 ± 0.219	0.092 ± 0.101
	Butanoic acid	6.150	0.072 ± 0.002			0.107 ± 0.002	
	Hexanoic acid	13.642	2.286 ± 0.265	1.000 ± 0 .608	1.393 ± 0.209	1.97 ± 0.073	1.486 ± 0.416
	α-Hydroxyisocaproic acid	15.102			0.008 ± 0.00	0.023 ± 0.006	0.027 ± 0.003
	Heptanoic acid	16.866	0.088 ± 0.004	0.051 ± 0.046	0.09 ± 0.043	0.037 ± 0.015	0.125 ± 0.046
	cis-5-Dodecenoic acid	30.154	0.022 ± 0.009	0.036 ± 0.015	0.051 ± 0.041	0.102 ± 0.041	0.135 ± 0.064
	Z-11-Tetradecenoic acid	34.855		0.015 ± 0.009	0.027 ± 0.001	0.041 ± 0.02	0.041 ± 0.026
	Pentanoic acid, 2-methyl-	36.992	0.028 ± 0.007		0.033 ± 0.004		
	Linoelaidic acid	43.239	0.162 ± 0.111	0.305 ± 0.167	0.334 ± 0.042	0.568 ± 0.401	0.176 ± 0.032
	9,12-Octadecadienoic acid (Z,Z)-	43.258					0.696 ± 0.169
	Octanoic acid	43.429			0.063 ± 0.045		
**Σ(Type)**			6	5	9	8	8
**Σ(Concentration)**			2.658	1.407	2.078	3.118	2.779
**Alcohols (7)**	2,3-Butanediol, [R-(R*,R*)]-	6.345				0.644 ± 0.995	
	2,3-Butanediol	7.345				0.215 ± 0.177	
	1-Hexanol	8.165					0.087 ± 0.008
	1-Dodecanol	14.395			0.017 ± 0.005	0.03 ± 0.004	
	Phenylethyl Alcohol	16.930	0.06 ± 0.002	0.125 ± 0.008	0.128 ± 0.009	0.218 ± 0.079	0.254 ± 0.066
	1-Octanol, 2-butyl-	27.074			0.031 ± 0.016		
	Z-11-Pentadecenol	34.956	0.071 ± 0.079				

**Σ(Type)**			2	1	3	4	2
**Σ(Concentration)**			0.108	0.125	0.176	1.107	0.341
**Ketones (3)**	2,4,6-Cycloheptatrien-1-one, 4-methyl-	14.611	0.29 ± 0.013				
	2-Nonanone	16.130	0.011 ± 0.003	0.014 ± 0.003	0.02 ± 0.003	0.022 ± 0.003	0.013 ± 0.001
	2-Tridecanone	34.954				0.009 ± 0.002	0.011 ± 0.008
**Σ(Type)**			2	1	1	2	2
**Σ(Concentration)**			0.301	0.014	0.020	0.032	0.024
**Aldehyde (3)**	Furfural	6.530	0.132 ± 0.03				
	Nonanal	16.770	0.099 ± 0.035		0.032 ± 0.02	0.026 ± 0.004	
	Decanal	19.955		0.025 ± 0.003	0.014 ± 0.01		
**Σ(Type)**			2	1	2	1	0
**Σ(Concentration)**			0.230	0.025	0.047	0.026	0.000
**Phenols (1)**	2-Methoxy-4-vinylphenol	23.277	0.022 ± 0.011				
**Σ(Type of all volatiles)**			41	31	46	46	41
**Σ(Concentration of all volatiles)**			9.364	12.669	15.383	22.484	23.590

Empty represents that the substance was not detected. The data with orange background indicates that the substance in the sample has increased by more than 5% compared to the sample at the previous time point.

Table S2. High quality sequence based on 16S rRNA and ITS sequencing.

Samples	No. of sequences				Proportion (%)	
	Effective of sequence		High-quality sequences			
	**Bac.**	**Fun.**	**Bac.**	**Fun.**	**Bac.**	**Fun.**
d0_1	48707	66240	47936	65899	98.42%	99.49%
d0_2	41364	92135	40770	91736	98.56%	99.57%
d0_3	45557	59354	45284	59182	99.40%	99.71%
d7_1	52291	79938	51450	79919	98.39%	99.98%
d7_2	53713	62037	52712	62022	98.14%	99.98%
d7_3	48662	64676	47657	64667	97.93%	99.99%
d14_1	42963	64016	42596	63989	99.15%	99.96%
d14_2	43776	65797	43666	65775	99.75%	99.97%
d14_3	42091	61893	42024	61881	99.84%	99.98%
d21_1	43908	66230	43480	66214	99.03%	99.98%
d21_2	46333	74173	46110	74149	99.52%	99.97%
d21_3	43450	36443	43336	36432	99.74%	99.97%
d28_1	73178	52959	72343	52937	98.86%	99.96%
d28_2	63897	57129	63718	57089	99.72%	99.93%
d28_3	55707	53043	55580	52975	99.77%	99.87%

Table S3. The microbial community richness and diversity indices for microbial communities based on 16SrRNA and ITS sequencing.

**Samples**		**OTUs**	Diversity index		Abundance index		Coverage
			Shannon	Simpson	Ace	Chao	
**Bacteria**	d0_1	272	3.5192	0.0608	275.2494	274.6190	0.9997
	d0_2	251	3.2231	0.0867	254.7229	253.8696	0.9997
	d0_3	213	2.8782	0.1467	215.3461	217.5000	0.9998
	d7_1	39	0.7749	0.5907	43.1938	41.3333	0.9998
	d7_2	24	0.7849	0.5300	25.4694	24.6000	0.9999
	d7_3	30	0.9221	0.4538	35.5177	39.3333	0.9998
	d14_1	6	0.1715	0.9228	6.4160	6.0000	1.0000
	d14_2	8	0.0219	0.9940	9.0909	8.3333	1.0000
	d14_3	5	0.0096	0.9977	12.7333	6.0000	1.0000
	d21_1	7	0.1857	0.9170	14.7333	8.0000	1.0000
	d21_2	5	0.0812	0.9712	5.0000	5.0000	1.0000
	d21_3	12	0.0268	0.9939	12.0000	12.0000	1.0000
	d28_1	7	0.3107	0.8365	13.7800	8.0000	1.0000
	d28_2	13	0.0365	0.9906	14.5447	13.2500	1.0000
	d28_3	9	0.0167	0.9960	10.1313	9.5000	1.0000
**Fungi**	d0_1	85	1.8807	0.2085	102.5780	102.2727	0.9995
	d0_2	86	1.9190	0.2173	92.8181	90.7143	0.9997
	d0_3	81	1.8266	0.2292	88.5846	87.0000	0.9997
	d7_1	29	0.1607	0.9565	32.4630	29.7500	0.9999
	d7_2	28	0.1074	0.9724	35.6317	32.2000	0.9998
	d7_3	27	0.1669	0.9540	42.3754	34.0000	0.9998
	d14_1	48	0.4535	0.8418	50.0411	49.4286	0.9999
	d14_2	57	0.3838	0.8797	70.6024	68.6667	0.9996
	d14_3	33	0.2448	0.9264	41.1182	36.5000	0.9998
	d21_1	38	0.4310	0.8415	43.5053	42.2000	0.9998
	d21_2	49	0.5045	0.8152	50.9073	49.7500	0.9999
	d21_3	36	1.4927	0.3633	38.0327	37.0000	0.9999
	d28_1	41	1.1467	0.5033	46.6462	42.0000	0.9999
	d28_2	37	1.4480	0.3308	38.4429	38.0000	0.9999
	d28_3	37	1.1063	0.5034	37.3945	37.0000	1.0000

Table S4. The enzymes related to ester formation of microbial community in FGs predicted by PICRUSt2 and their annotation.

Enzyme number	Enzyme category
EC 3.2.1.3	Glucan 1,4-alpha-glucosidase
EC 3.2.1.21	Beta-glucosidase
EC 3.2.1.4	Cellulase
EC 2.7.1.2	Glucokinase
EC 2.7.1.1	Hexokinase
EC 4.2.1.11	Phosphopyruvate hydratase
EC 2.7.1.40	Pyruvate kinase
EC 1.2.4.1	Pyruvate dehydrogenase (acetyl-transferring)
EC 1.8.1.4	Dihydrolipoyl dehydrogenase
EC 2.3.1.12	Dihydrolipoyllysine-residue acetyltransferase
EC 1.2.7.1	Pyruvate synthase
EC 1.2.7.11	2-oxoacid oxidoreductase (ferredoxin)
EC 1.1.1.27	L-lactate dehydrogenase
EC 1.1.1.28	D-lactate dehydrogenase
EC 1.2.1.3	Aldehyde dehydrogenase (NAD(+))
EC 1.2.1.5	Aldehyde dehydrogenase (NAD(P)(+))
EC 1.1.1.35	3-hydroxyacyl-CoA dehydrogenase
EC 1.1.1.36	Acetoacetyl-CoA reductase
EC 1.1.1.157	3-hydroxybutyryl-CoA dehydrogenase
EC 2.3.1.16	Acetyl-CoA C-acyltransferase
EC 2.3.1.8	Phosphate acetyltransferase
EC 2.3.1.9	Acetyl-CoA C-acetyltransferase
EC 2.7.2.1	Acetate kinase
EC 2.7.2.7	Butyrate kinase
EC 2.8.3.8	Acetate CoA-transferase
EC 4.2.1.55	3-hydroxybutyryl-CoA dehydratase
EC 4.2.1.7	Altronate dehydratase
EC 1.2.1.10	Acetaldehyde dehydrogenase (acetylating)
EC 3.1.2.1	Acetyl-CoA hydrolase
EC 6.2.1.1	Acetate--CoA ligase
EC 6.2.1.13	Acetate--CoA ligase (ADP-forming)
EC 2.8.3.18	Succinyl-CoA:acetate CoA-transferase
EC 1.3.1.86	Crotonyl-CoA reductase
EC 1.3.1.44	Trans-2-enoyl-CoA reductase (NAD(+))
EC 2.3.1.19	Phosphate butyryltransferase
EC 4.2.1.17	Enoyl-CoA hydratase
EC 1.3.1.38	Trans-2-enoyl-CoA reductase (NADPH)
EC 4.1.1.1	Pyruvate decarboxylase
EC 1.1.1.1	Alcohol dehydrogenase
EC 1.1.1.2	Alcohol dehydrogenase (NADP(+))
EC 1.1.2.7	Methanol dehydrogenase (cytochrome c)
EC 1.1.2.8	Alcohol dehydrogenase (cytochrome c)
EC 3.1.1.1	Carboxylesterase
EC 3.1.1.23	Acylglycerol lipase
EC 3.1.1.3	Triacylglycerol lipase

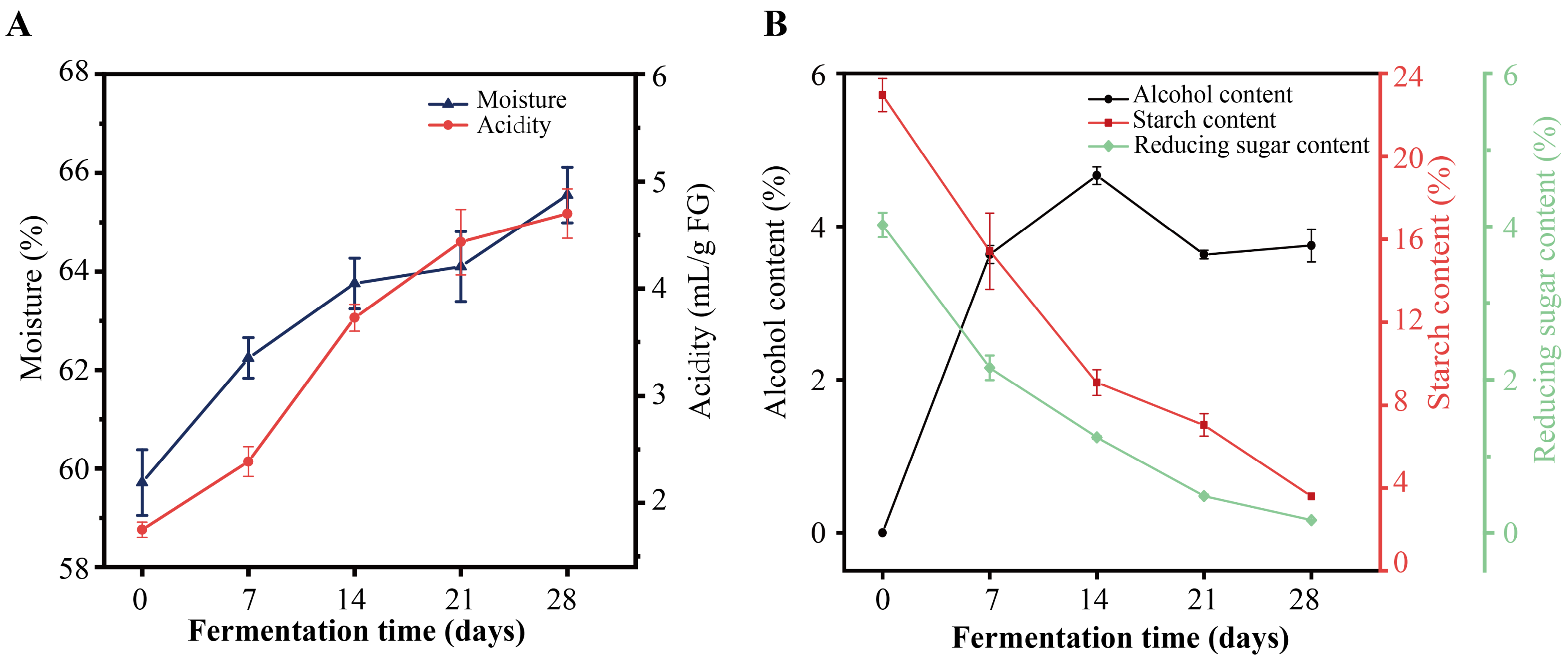
A
Moisture
Acidity
Moisture (%)
Acidity (mL/g FG)
Fermentation time (days)
B
Alcohol content
Starch content
Reducing sugar content
Alcohol content (%)
Starch content (%)
Reducing sugar content (%)
Fermentation time (days)

Figure S1

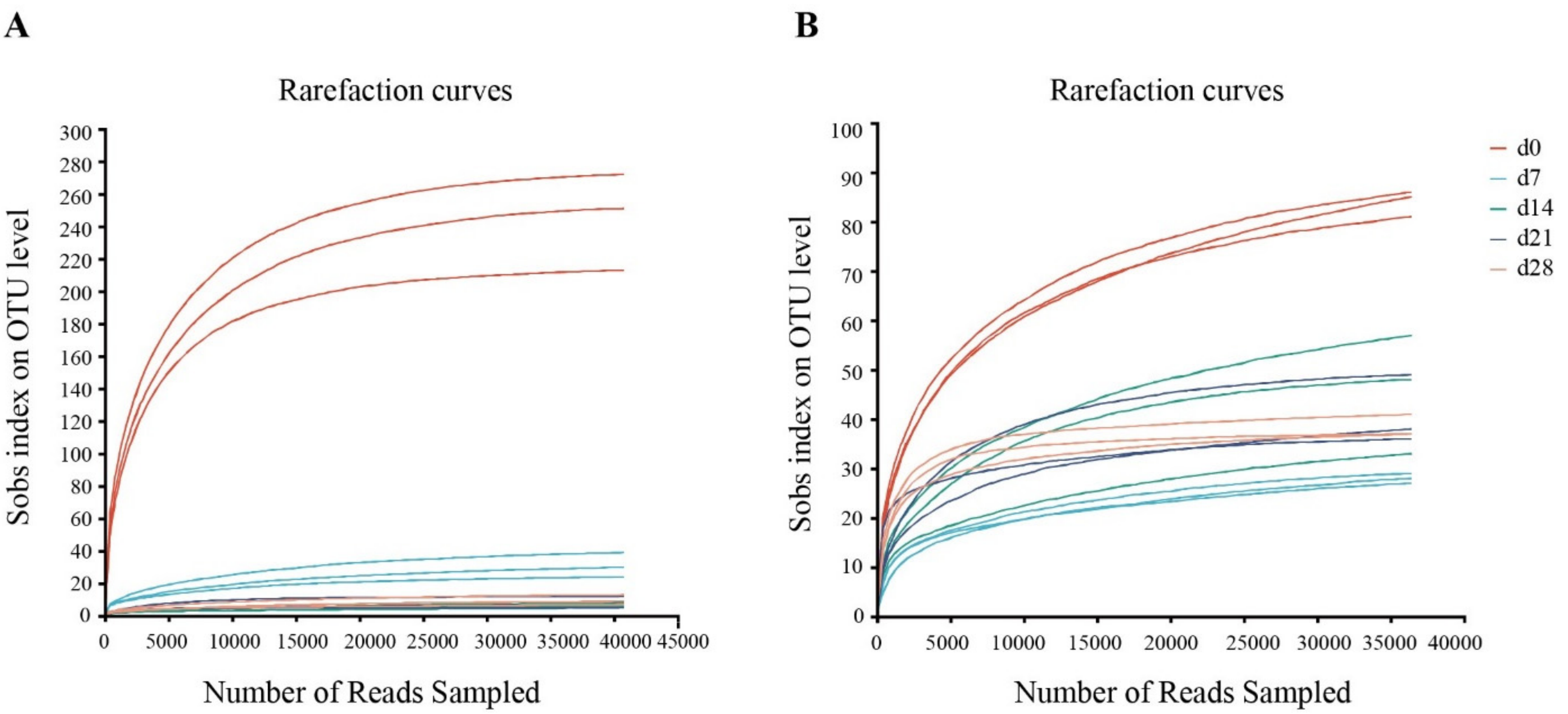
A
Rarefaction curves
Sobs index on OTU level
Number of Reads Sampled
0
20
40
60
80
100
120
140
160
180
200
220
240
260
280
300
5000
10000
15000
20000
25000
30000
35000
40000
45000
B
Rarefaction curves
Sobs index on OTU level
Number of Reads Sampled
10
30
50
70
90
d0
d7
d14
d21
d28

Figure S2

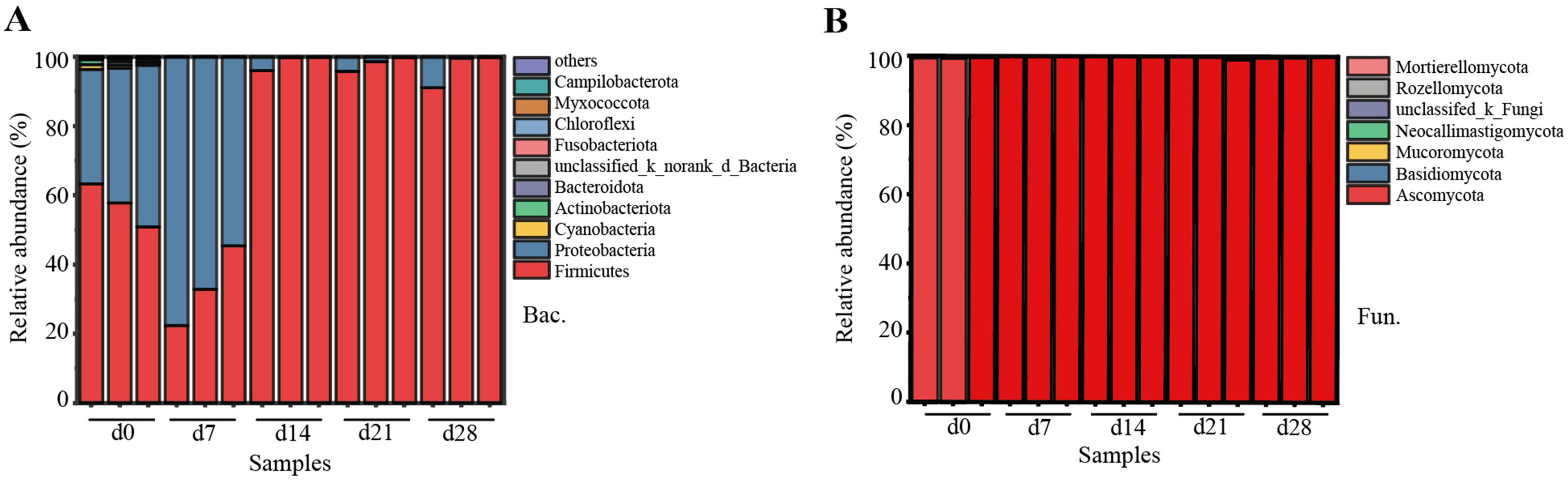
A
Relative abundance (%)
0
20
40
60
80
100
d0
d7
d14
d21
d28
Samples
others
Campilobacterota
Myxococcota
Chloroflexi
Fusobacteriota
unclassified_k_norank_d_Bacteria
Bacteroidota
Actinobacteriota
Cyanobacteria
Proteobacteria
Firmicutes
Bac.
B
Relative abundance (%)
0
20
40
60
80
100
d0
d7
d14
d21
d28
Samples
Mortierellomycota
Rozellomycota
unclassifed_k_Fungi
Neocallimastigomycota
Mucoromycota
Basidiomycota
Ascomycota
Fun.

Figure S3

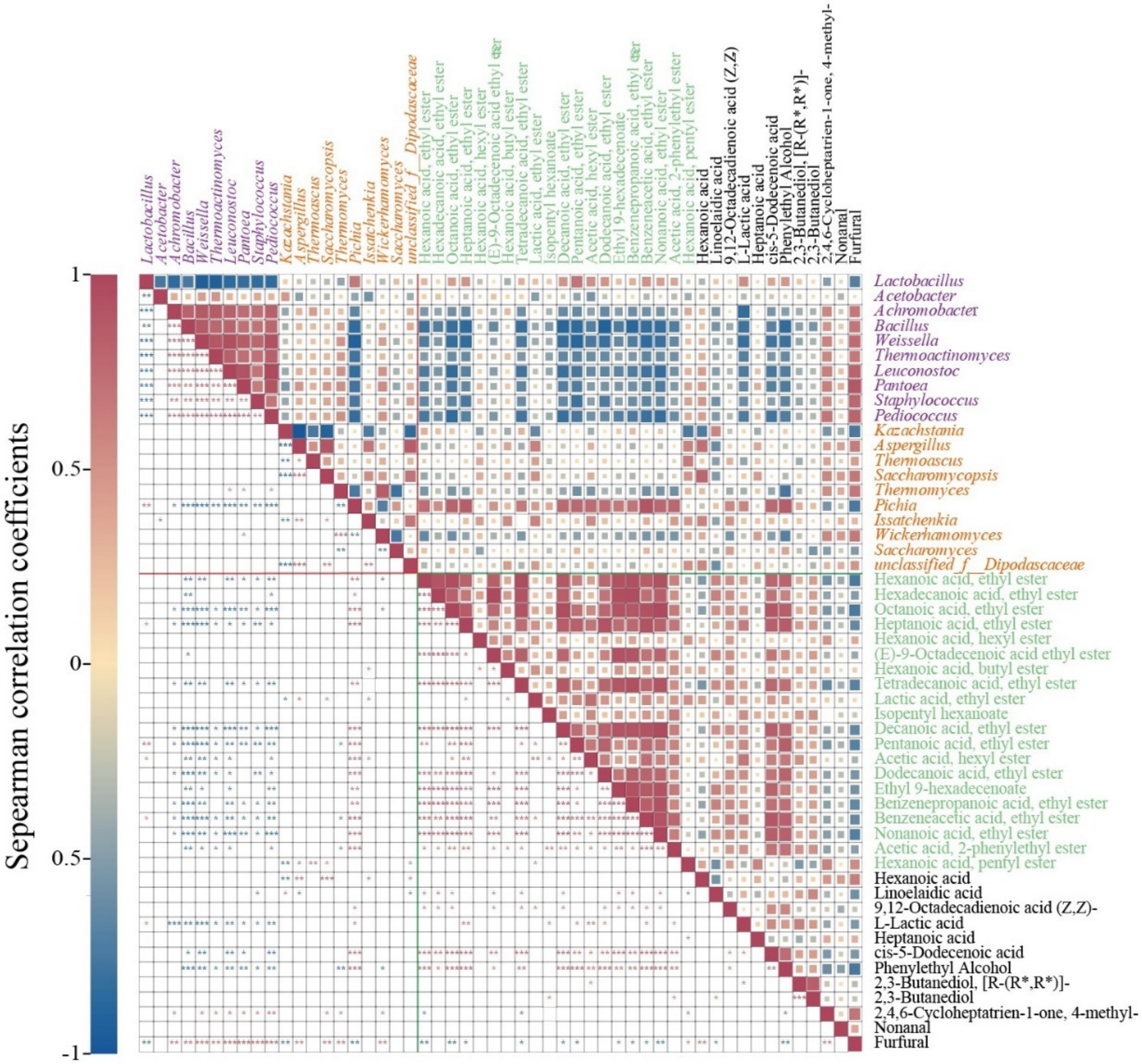
Sepearman correlation coefficients
1
0.5
0
0.5
-1
Lactobacillus
Acetobacter
Achromobacter
Bacillus
Weissella
Thermoactinomyces
Leuconostoc
Pantoea
Staphylococcus
Pediococcus
Kazachstania
Aspergillus
Thermoascus
Saccharomycopsis
Thermomyces
Pichia
Issatchenkia
Wickerhamomyces
Saccharomyces
unclassified_f__Dipodascaceae
Hexanoic acid, ethyl ester
Hexadecanoic acid, ethyl ester
Octanoic acid, ethyl ester
Heptanoic acid, ethyl ester
Hexanoic acid, hexyl ester
(E)-9-Octadecenoic acid ethyl ester
Hexanoic acid, butyl ester
Tetradecanoic acid, ethyl ester
Lactic acid, ethyl ester
Isopentyl hexanoate
Decanoic acid, ethyl ester
Pentanoic acid, ethyl ester
Acetic acid, hexyl ester
Dodecanoic acid, ethyl ester
Ethyl 9-hexadecenoate
Benzenepropanoic acid, ethyl ester
Benzeneacetic acid, ethyl ester
Nonanoic acid, ethyl ester
Acetic acid, 2-phenylethyl ester
Hexanoic acid, pentyl ester
Hexanoic acid
Linoelaidic acid
9,12-Octadecadienoic acid (Z,Z)-
L-Lactic acid
Heptanoic acid
cis-5-Dodecenoic acid
Phenylethyl Alcohol
2,3-Butanediol, [R-(R*,R*)]-
2,3-Butanediol
2,4,6-Cycloheptatrien-1-one, 4-methyl-
Nonanal
Furfural

Figure S4
